# Assessing the inter-rater reliability of the Schizophrenia Cognition Rating Scale: a non-interventional quantitative study

**DOI:** 10.1038/s41537-025-00619-9

**Published:** 2025-04-28

**Authors:** Sebastien Tulliez, Stella Karantzoulis, James C. Marcus, Montserrat Casamayor, Cassie Blanchard, Haig Goenjian, Joshua T. Kantrowitz, Lara Shirikjian, John Sonnenberg, Corey Reuteman-Fowler, Philip D. Harvey, Richard S. E. Keefe

**Affiliations:** 1 Boehringer Ingelheim International GmbH, Ingelheim am Rhein, Germany; 2 IQVIA, New York, NY USA; 3 IQVIA, Washington, DC USA; 4 IQVIA, Provença, 392 Barcelona, Spain; 5 CenExel Hassman Research Institute, Berlin, NJ USA; 6 CenExel Collaborative Neuroscience Research, Garden Grove, CA USA; 7 New York State Psychiatric Institute, New York, NY USA; 8 Columbia University, College of Physicians and Surgeons, New York, NY USA; 9 Nathan Kline Institute, Orangeburg, NY USA; 10 CenExel Collaborative Neuroscience Research, Torrance, CA USA; 11 Uptown Research Institute, Chicago, IL USA; 12 Northwestern University Feinberg School of Medicine, Chicago, IL USA; 13 Boehringer Ingelheim Pharmaceuticals, Inc., Ridgefield, CT USA; 14 University of Miami Miller School of Medicine, Miami, FL USA; 15 Duke University Medical Center, Durham, NC USA

**Keywords:** Schizophrenia, Psychosis

## Abstract

Background: Cognitive impairment is a core feature of schizophrenia, profoundly impacting patients’ functional abilities. As such, evaluating cognition-related functional activity/impairment is essential for identifying effective treatments. This study presents findings from a non-interventional quantitative study to assess the inter-rater reliability (IRR) of the Schizophrenia Cognition Rating Scale (SCoRS) with a sample representative of clinical trial populations. Methods: Structured, one-to-one, 10–15-minute live interviews with patients with schizophrenia were conducted by trained SCoRS interviewers (raters), and a separate interview was then conducted with the patient’s study partner (informant). Both interviews were recorded so that each interview was assessed by three different SCoRS raters in total (one live, two via recording). IRR was assessed using interclass correlation (ICC) and categorized as low (<0.70), good (0.70–0.90), or excellent (>0.90). Results: A total of 44 patients with schizophrenia were evaluated by 12 raters (overall). The SCoRS Total Score (mean [SD]: 41.4 [10.2]) indicated moderate-to-moderately-severe impairment of cognition-related functioning, with high inter-patient variability. The SCoRS Total Score demonstrated excellent IRR, with an ICC of 0.91 (95% CI 0.88–0.95). Conclusion: The 20-item SCoRS Total Score demonstrated excellent IRR in assessing cognition-related functional capacity in patients with schizophrenia, supporting its use as an endpoint in clinical studies.

## Introduction

Cognitive impairment is a core feature of schizophrenia, profoundly impacting the functional abilities of people living with the condition^1,2^. This impairment contributes to disability in everyday life, unemployment, and substantial challenges to autonomous daily living^2–4^. As such, assessing cognitive impairment and its impact on functional activity is essential in patients with schizophrenia^5^. Although there are many cognitive rating scales, their relative merits are unclear and cognitive impairment associated with schizophrenia (CIAS) is conventionally assessed using performance-based cognitive measures, such as the Measurement and Treatment Research to Improve Cognition in Schizophrenia (MATRICS) Consensus Cognitive Battery (MCCB) or the Brief Assessment of Cognition in Schizophrenia (BACS)^6–8^.

Currently, there are no approved pharmacological treatments for cognitive impairment associated with schizophrenia, though new treatments with novel mechanisms of action are in development^9,10^. The United States Food and Drug Administration (FDA) is actively collaborating with researchers to address this unmet need and has expressed concern over the face validity of performance-based measures in evaluating treatments for schizophrenia^11,12^. To address this, Keefe et al. (2006) developed the Schizophrenia Cognition Rating Scale (SCoRS), an interview-based assessment of cognition-related functional capacity^13^. The initial version consisted of 18 items, but was subsequently modified to remove an item on motor functioning and add three items on social cognition, aligning it with the cognitive domains of the MCCB and BACS^7,8^. The 20-item SCoRS has since demonstrated strong psychometric properties, with publications in 2015 reporting excellent test-retest reliability, convergence with cognitive performance, and sensitivity to treatment^14,15^, and is increasingly used as an outcome measure in randomized clinical trials to measure cognitive improvement over time^15–20^.

A critical prerequisite for any rater-based assessment is understanding inter-rater reliability (IRR), i.e., the degree to which trained raters would agree when assessing the same patient using the same measure. While a previous study assessed the IRR of the 18-item SCoRS^13^, it had several limitations. Firstly, it was conducted with only a small population of 11 inpatients at a rehabilitation center, all with fairly severe cognitive impairment, which restricted the range of scores and possibly inflated the IRR estimate. Secondly, all patients were evaluated by the same two raters who conducted each interview jointly. This atypical joint interview format may have artificially decreased between-rater variability and introduced rater-specific idiosyncrasies or biases. Thirdly, informants were rehabilitation center staff, meaning they were better trained in schizophrenia than typical informants and were not unique to each patient. Finally, the 18-item SCoRS was assessed, not the 20-item version, and IRR was not reported for SCoRS Total Score, only at the item-level. Additionally, while IRR was reported using agreement-based intraclass correlations (ICCs), the estimates lacked confidence intervals (CIs)^13^. It is important that even the lower bound of the IRR estimate exceeds a desired threshold, ensuring the measure’s reliability is beyond doubt.

The objective of this study was to expand on previous studies, by evaluating the IRR of the 20-item SCoRS Total Score in a larger and more diverse population of patients with schizophrenia. In line with this, the study employed a robust design and included patients with a wider range of characteristics and levels of functional cognitive impairment, aiming to make the findings more applicable to both clinical trial populations and people with schizophrenia more broadly.

## Results

### Study population

In total, 50 patients were recruited into the study across the five sites. Of these, 44 patients (and 44 corresponding informants) underwent live interviews with one of the 12 different SCoRS raters participating in the study; reasons for excluding six patients from the study are summarized in Supplementary Table 1. Of note, nearly half (*n* = 20; 45.5%) of all patients were recruited at one study site (#5) due to the availability of eligible patients and recruitment challenges at other sites, and had their live interview with one of two SCoRS raters at this site.

### Demographics and baseline characteristics

Demographics and baseline characteristics for patients, informants, and SCoRS raters are presented in Tables 1–3, respectively. Overall, patient demographics and characteristics were consistent with a schizophrenia clinical trial population. Most (*n* = 8; 66.7%) SCoRS raters had at least 10 years’ experience managing/treating patients with schizophrenia, and most informants were either family (*n* = 20; 45.5%) or a friend (*n* = 20; 45.5%).

**Table 1:** Patient demographics and baseline characteristics.

	All patients (*N* = 44)
**Age, median years (IQR)**	39 (33.5–44.5)
**Male, n (%)**	28 (63.6)
**Race, n (%)**	
White	14 (31.8)
Non-white	30 (68.2)
**Living status, n (%)**	
Alone	12 (27.3)
With partner	4 (9.1)
With parents	8 (18.2)
With other family members	6 (13.6)
With roommates	6 (13.6)
In residential care	2 (4.5)
In another supported care environment	3 (6.8)
Not listed	3 (6.8)
**Work status, n (%)**	
Full-time employment	3 (6.8)
Part-time employment	5 (11.4)
Freelance/contractor	2 (4.5)
Student	2 (4.5)
Unable to work due to condition	22 (50.0)
Unemployed (unrelated to condition)	9 (20.5)
Not listed	1 (2.3)
**Highest education level, n (%)**	
High school (or less)	24 (54.5)
High school to Bachelor’s degree	12 (27.3)
Bachelor’s degree	6 (13.6)
Advanced degree	1 (2.3)
Other	1 (2.3)
**Time since diagnosis, median years (IQR)**	13.7 (8.8–23.0)

*IQR* interquartile range.

**Table 2:** Informant demographics and baseline characteristics.

	All informants (*N* = 44)
**Age, median years (IQR)**	54 (37.5–61.5)
**Male, n (%)**	24 (54.5)
**Relation to patient, n (%)**	
Family	20 (45.5)
Friend	20 (45.5)
Other	4 (9.1)
**Highest education level, n (%)**	
High school (or less)	22 (50.0)
High school to Bachelor’s degree	12 (27.3)
Bachelor’s degree	8 (18.2)
Advanced degree	1 (2.3)
Other	1 (2.3)
**Total contact with patient, h/week, n (%)**	
1	0
2–4	10 (22.7)
5–10	6 (13.6)
>10	28 (63.6)
**Average face-to-face contact with patient, h/week, n (%)**	
1	1 (2.3)
2–4	9 (20.5)
5–10	9 (20.5)
>10	25 (56.8)

*IQR* interquartile range. *h* hours.

**Table 3:** Rater demographics and baseline characteristics.

	All raters (*N* = 12)
**Male, n (%)**	3 (25.0)
**Current role, n (%)**	
Rater	5 (41.7)
Assistant research scientist	1 (8.3)
Psychometric rater	1 (8.3)
Rater/sub-investigator	1 (8.3)
Research scientist	1 (8.3)
Senior clinical rater	1 (8.3)
Senior rater	1 (8.3)
Clinical research nurse	1 (8.3)
**Years managing/treating patients with schizophrenia, n (%)**	
3–4	1 (8.3)
5–10	3 (25.0)
>10	8 (66.7)
**Average patients with schizophrenia treated/month, n (%)**	
None	2 (16.7)
11–20	4 (33.3)
21–50	3 (25.0)
>50	3 (25.0)
**Number of previous SCoRS performed, n (%)**	
None	1 (8.3)
5–10	2 (16.7)
11–20	1 (8.3)
21–50	3 (25.0)
>50	5 (41.7)
**Time since last SCoRS training, median years (IQR)**	1.2 (0.7–1.5)

*IQR* interquartile range, *SCoRS* the Schizophrenia Cognition Rating Scale.

### Inter-rater reliability

**Fig. 1:**
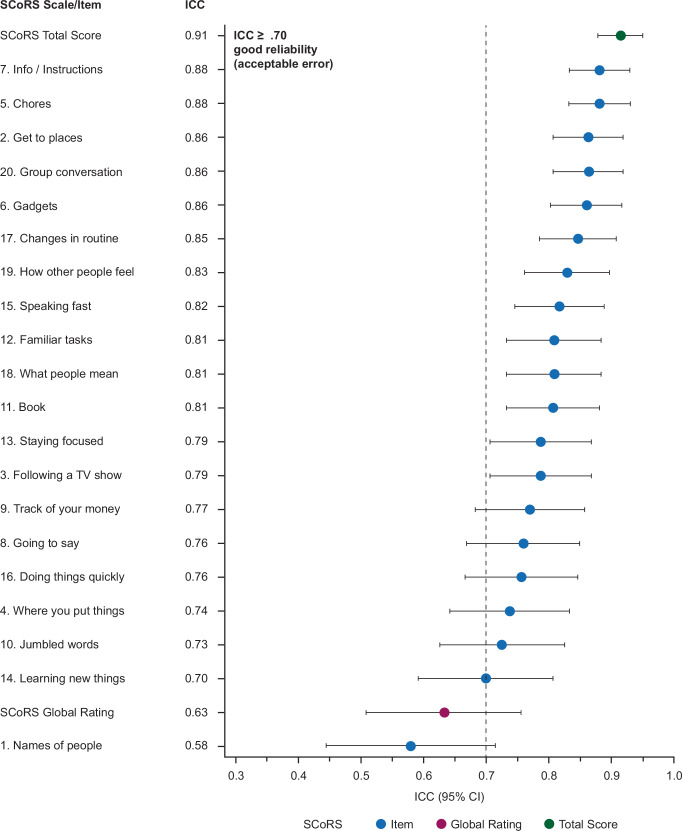
ICCs for the SCoRS Total Score, the SCoRS Global Rating Score, and the individual SCoRS items by descending ICC value. CI confidence interval, ICC interclass correlation, SCoRS the Schizophrenia Cognition Rating Scale.

The SCoRS Total Score demonstrated excellent IRR, with an ICC of 0.91 (95% CI 0.88–0.95), both greater than the predicted ICC (0.82) and consistent with the item-level results reported in the previous reliability analysis of the 18-item SCoRS (ICC > 0.9 for all items)^13^. In addition, the lower ICC 95% CI boundary for the SCoRS Total Score (0.88) was also substantially greater than the minimum desired (≥0.70). The individual SCoRS items and the Global Rating Score showed a similar trend, with all but one item having ‘good’ IRR (an ICC of 0.70–0.90), and 13 of the 20 also having lower 95% CI bounds >0.70 (Fig. 1). The Global Rating Score had ‘low’ IRR (ICC < 0.70).

As 45.5% of all patients were recruited at a single study site (#5), a *post hoc* sensitivity analysis was conducted to determine if ICC values based on interviews conducted at the site differed from those based on interviews conducted at the other four sites. The ICCs for the SCoRS Total Score were similar when live interviews were conducted at site #5 (0.91 [95% CI 0.86–0.97]) compared to when they were conducted at other sites (0.87 [95% CI 0.80–0.94]), as were all but one of the ICCs for the individual SCoRS items (Supplementary Table 2). The one exception was the ‘Names of people’ item, which indicated a higher ICC of 0.76 (95% CI 0.63–0.89) for site #5 compared with the rest of the study population, which had an ICC of 0.40 (95% CI 0.19–0.62).

### The SCoRS Total Score and Global Rating Score

Overall, the mean (SD) SCoRS Total Score was 41.4 (10.2; range 22–68). This value was consistent across all three interview modes (live interview, video recording 1 and video recording 2; mean values of 41.0–42.0; Supplementary Table 3). A similar level of cognition-related impairment of functional capacity was indicated by the SCoRS Global Rating Score, with a mean (SD) score of 4.5 (1.6) that was also consistent across all three interview modes (Supplementary Table 3).

### The SCoRS item response distributions

**Fig. 2:**
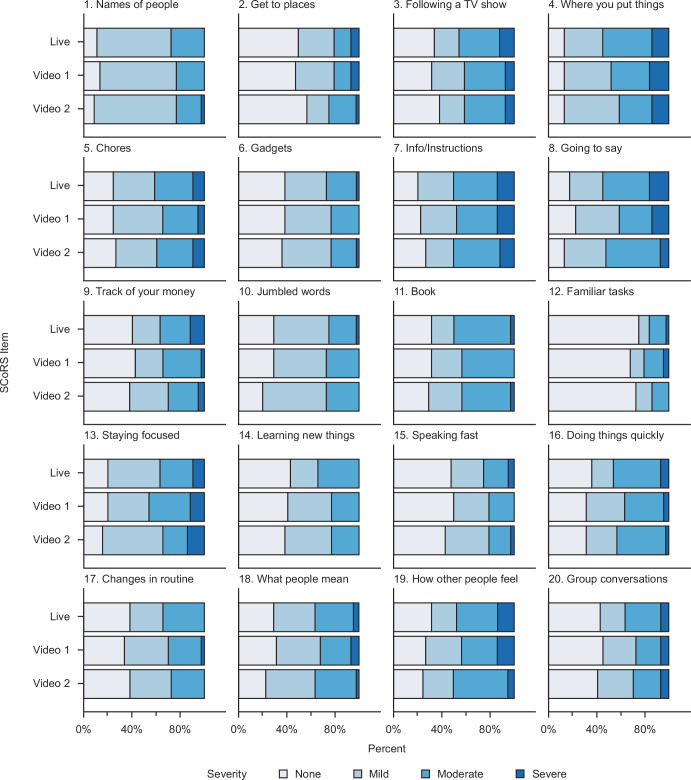
Response distributions for each of the individual SCoRS items. SCoRS the Schizophrenia Cognition Rating Scale.

The distribution of responses (‘none’, ‘mild’, ‘moderate’, ‘severe’) for each of the 20 SCoRS items is summarized in Fig. 2. Overall, responses were varied between patients and typically covered the whole range of response options. Distributions were also similar across items and rating modes (live, video 1, video 2); only items 1, 2 and 12 (‘Names of people’, ‘Get to places’, and ‘Familiar tasks’, respectively) showed a lack of variability, with a single response category accounting for >50% of patient responses (‘mild’ for item 1, and ‘none’ for items 2 and 12).

## Discussion

The SCoRS is an interview-based measure of cognition-related functional activity, which was developed for use as a measure of cognitive treatment response in clinical trials of schizophrenia^14^. This non-interventional, US-based, multicenter study aimed to evaluate the IRR of the 20-item version of the SCoRS via live, one-to-one interviews with both patients and their informants, along with separate assessments of the interviews via video recordings (each patient was rated by three different trained SCoRS raters). This was the first assessment of the reliability of SCoRS in a typical schizophrenia clinical trial population and demonstrated that the SCoRS Total Score had an excellent IRR, with an ICC of 0.91 and a lower 95% CI boundary of 0.88. This was consistent with the previous, more limited, evaluation of the 18-item SCoRS (IRR > 0.90)^13^, supporting the validity and generalizability of the findings.

The SCoRS developers emphasize that the SCoRS Total Score should be prioritized over the individual item scores as a more robust measure of cognition-related functional capacity^14^. Further, this study was not designed to assess differences in the IRR of individual items. Thus, it is notable that all but one item had ‘good’ IRR (an ICC of 0.70–0.90), with 13 of the 20 also having lower 95% CI bounds >0.70. The remaining item (#1: ‘Remembering names of people you know or meet’) had a low ICC (0.58), but sensitivity analyses showed the ICC was greater (0.76) for this item at the single study site (#5) where most patients were recruited. The low ICC may be partly explained by the limited between-patient variability for this item, with most patients being rated ‘Mild’. Another contributing factor may be that most informants were family or friends, who may have only considered the difficulty patients had in remembering their names or the names of other family members or friends with whom the patient interacts regularly. Similar results were observed in earlier assessments of the 20-item SCoRS, where memory-related items (‘Remembering information and/or instructions recently given to you’ and ‘Remembering what you were going to say’) also had some of the lowest test-retest reliability^14^, further indicating it may be inherent to patient/informant variability for the individual item.

Another similarity to the earlier SCoRS assessments was the lower IRR of the SCoRS Global Rating Score (ICC = 0.63) compared with the SCoRS Total Score^14^. This likely reflects the increased variability when relying on single scores compared with summated values, again highlighting the robustness of the SCoRS Total Score and supporting previous recommendations that this should be the primary SCoRS measure used in clinical trials^14^.

As an interview-based assessment, the SCoRS offers flexibility in how questions are posed, making it easier to adapt for international use compared with performance-based measures^8,21^. Currently available across 22 languages, the SCoRS has been used in several different countries^8,22–25^. Additionally, it imposes a very low burden on patients, informants, and raters^8^. However, it is important to note that informant interviews are a critical component of the SCoRS; in situations where patients lack a suitable informant, performance-based measures may be more suitable^8^. The SCoRS has been shown to strongly correlate with everyday functioning^8,13,26^. For a more detailed discussion of the evaluation, validation and implementation of the SCoRS, the reader is invited to refer to the review article by Harvey et al. (2019)^8^.

As with all studies, methodological limitations should be considered when interpreting the results. One limitation was the distribution of the patient population across study sites (and therefore across different raters), with nearly half of the patients recruited from a single study site. However, a sensitivity analysis showed that, except for item #1, all ICC values were consistent (with overlapping 95% CIs) between that study site and the other four, indicating that neither drove the primary results. Secondly, while the study aimed to include patients with a range of functional impairment scores to assess the IRR in a large and ecologically valid population, each patient’s SCoRS rating was not available until after the SCoRS interviews were complete. As such, it was not possible to incorporate these quotas at screening, so a proxy assessment was used instead. Nevertheless, the mean SCoRS Total Score was 41.4 (range 22–68), indicating moderate-to-moderately-severe impairment overall, consistent with expectations for a clinical trial of patients with cognitive impairment associated with schizophrenia. A wide range of scores was also observed across most items, with response options generally evenly distributed, indicating a broad range of cognition-related function. The IRR was estimated by rating patients three times – once via a live interview and twice based on video recordings of their live interviews – rather than by live interviews conducted by separate clinicians during a contiguous period. While this limitation was due to the study’s limited geographical scope, it also avoided the difficulties associated with multiple patient interviews over a short period of time, such as learning effects (by the interviewees). While the sample size of 44 participants may be perceived as a limitation, it was determined a priori to ensure a sufficient sample to detect the expected ICC, and this was borne out via the small CIs obtained for the scores. While this study establishes strong IRR for SCoRS within a controlled research setting (i.e., patients with a requisite degree of symptom and treatment stability), further research is always beneficial for evaluating its validity and generalizability to patients who would not meet minimal criteria for participation in a clinical trial.

In conclusion, results from this non-interventional quantitative study demonstrated that the 20-item SCoRS Total Score has excellent IRR when used to assess the cognition-related functional capacity of patients with schizophrenia. These IRR findings add to the body of evidence demonstrating the reliability, validity, and treatment sensitivity of the SCoRS^14^, and support the use of the 20-item measure as an endpoint in multicenter clinical studies of patients with cognitive impairment and schizophrenia.

## Methods

### Study design

This was a non-interventional, quantitative, standalone study to assess the IRR of the 20-item SCoRS measure (i.e., the degree to which different raters agree when scoring the same patient using the SCoRS). Structured, one-to-one, 10–15-minute live interviews with patients with schizophrenia were conducted by trained SCoRS interviewers (raters). These interviews were conducted between September 28, 2022, and October 02, 2023, across five study sites in the US, each of which was required to provide at least two trained SCoRS raters.

### Study population

Patients eligible for interview met the following criteria: had a confirmed diagnosis of schizophrenia according to the Diagnostic and Statistical Manual of Mental Disorders, 5^th^ edition (DSM-V); had been on a stable regimen of antipsychotic medication for at least 12 weeks (up to two were permitted, excluding clozapine), maintaining their current dose for at least 35 days; functional impairment in day-to-day activities (e.g., conversational, focus, or memory difficulties); had not taken part in a SCoRS interview within the past 3 months; were deemed reliable and physiologically capable of participating in the SCoRS interview in the opinion of the study investigator; and had a suitable study partner (informant; e.g., a family member, friend, study nurse, or social worker) who knew the patient well and interacted with them regularly (for at least 1 h/week, ideally at least twice/week). Full inclusion and exclusion criteria are presented in Supplementary Table 4.

Eligible SCoRS raters were required to be medical professionals or experienced raters with at least 1 year of experience working with patients with schizophrenia and they needed to be fully qualified and trained in conducting SCoRS interviews by the WCG Clinical Endpoint Solutions (WCG™; the SCoRS instrument license holder). A summary of the rater requirements and training, and video-recording standards is provided in Supplementary Table 5.

### Data collection workflow

**Fig. 3:**
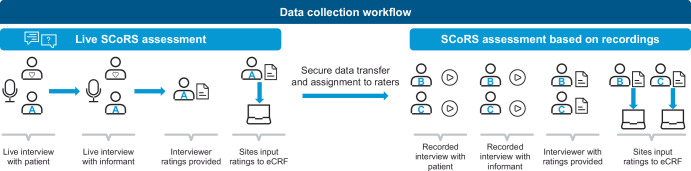
Study design overview. eCRF electronic case report form, SCoRS the Schizophrenia Cognition Rating Scale.

Within 7 days of the initial live patient interview, a separate 10–15-minute interview was conducted with each patient’s informant. Both the patient and informant SCoRS ratings were then completed and submitted by the rater (via electronic case report forms) within 48 h of the second interview. Due to various challenges with performing consecutive interviews with different raters to assess IRR (e.g., impracticality, learning effects), each live interview was video recorded. These recordings were then assessed independently by two additional SCoRS raters (Fig. 3), organized using a balanced incomplete block design (adapted for 12 raters) to ensure an even distribution of live and recorded interviews per rater.

### Ethical considerations

The study was conducted in accordance with all appropriate data confidentiality regulations and legislation, and all relevant institutional review board approvals (WIRB Copernicus Group) and informed patient and informant consent were obtained prior to study start and/or study participation.

### Statistical methodology

Sample size was determined based on an expected ICC of 0.82 (established using pilot study data [data on file]) and a desired CI range of 0.17 to ensure that even the lower bound would have acceptable error (i.e., an ICC with a lower CI bound of 0.65, meaning that at least ~70% of the variance would be due to patient differences and not rater differences / other sources of error)^27^. With three ratings per patient, this lower bound requirement resulted in the study needing to enroll 42 patients with schizophrenia, along with their informants.

For each of the 20 SCoRS items, one of the following responses could be recorded: ‘Not at all’ (1), ‘Mild’ (2), ‘Moderate’ (3), ‘Severe’ (4), or ‘Not applicable’. These responses were summed to yield a total score of 20–80. If more than five responses were ‘Not applicable’ or missing, then no total score was derived. The SCoRS also included a clinician-derived ‘Global Rating’ score from 0–10, with higher values indicating more severe impairment of cognition-related functioning. This clinician-assigned score reflects the overall severity of a patient’s cognitive impairment based on information gathered from the patient, their informant, and the clinician’s judgment.

All statistical analyses were performed using pooled data from across interview modes unless otherwise stated, using SAS Version Enterprise Guide 8.4 or higher (SAS Institute, North Carolina).

IRR was assessed via multiple-rater agreement-based ICC point estimates (ranging from 0–1) together with associated 95% CIs^28,29^. ICC values represent the proportion of variance attributable to between-patient differences, with <0.70 indicating ‘low’ IRR; 0.70–0.90, ‘good’ IRR; and >0.90, ‘excellent’ IRR^27^. ICCs were calculated for the SCoRS Total Score and for each individual SCoRS item using a null random effects model, with 95% CIs calculated using the delta method^30^.

## Supplementary information

Revised clean supplemental material

## Data Availability

IQVIA was contracted by Boehringer Ingelheim to conduct the analyses, interpret the results, as well as write, review, and revise the manuscript. To ensure independent interpretation of clinical study results and enable authors to fulfill their role and obligations under the ICMJE criteria, Boehringer Ingelheim grants all external authors access to clinical study data pertinent to the development of the publication. In adherence with the Boehringer Ingelheim Policy on Transparency and Publication of Clinical Study Data, scientific and medical researchers can request access to clinical study data when it becomes available on Vivli - Center for Global Clinical Research Data, and earliest after publication of the primary manuscript in a peer-reviewed journal, regulatory activities are complete, and other criteria are met. Please visit Medical & Clinical Trials | Clinical Research | MyStudyWindow for further information.
