# Supplementary material for: Assessing the inter-rater reliability of the Schizophrenia Cognition Rating Scale: a non-interventional quantitative study
Source: Schizophrenia (Heidelb). 2025 Apr 28;11(1):71. doi: 10.1038/s41537-025-00619-9 (PMC12037850; doi:10.1038/s41537-025-00619-9)
Supplement: Supplementary file 1 — Revised clean supplemental material [file 41537_2025_619_MOESM1_ESM.docx]

## **Supplementary materials**

#### Supplementary Table 1. Reasons for screen failure or protocol deviation.

| **Number of patients** | **Reason for screen failure or protocol deviation** |
| --- | --- |
| 1 | Did not meet PANSS inclusion criteria |
| 3 | Age (over 50 years) |
| 1 | Technical issues (no video) |
| 1 | Being on clozapine (an excluded medication) |

PANSS, Positive and Negative Syndrome Scale.

#### Supplementary Table 2. ICCs for the SCoRS Total Score, Global Rating Score, and individual items, stratified by site #5 (n=20) and the rest of the study population (sites #1–4; n=24).

| **SCoRS item** | **ICC [95% CI]** | |
| --- | --- | --- |
|  | **Sites #1–4** | **Site #5** |
| 1. Names of people | 0.40 [0.19–0.62] | 0.76 [0.63–0.89] |
| 2. Get to places | 0.80 [0.70–0.91] | 0.95 [0.91–0.98] |
| 3. Following a TV show | 0.73 [0.59–0.86] | 0.83 [0.74–0.93] |
| 4. Where you put things | 0.78 [0.67–0.89] | 0.67 [0.50–0.84] |
| 5. Chores | 0.85 [0.77–0.93] | 0.90 [0.84–0.96] |
| 6. Gadgets | 0.85 [0.76–0.93] | 0.87 [0.79–0.95] |
| 7. Info / Instructions | 0.83 [0.74–0.92] | 0.93 [0.88–0.97] |
| 8. Going to say | 0.69 [0.55–0.84] | 0.84 [0.74–0.93] |
| 9. Track of your money | 0.76 [0.64–0.88] | 0.79 [0.67–0.91] |
| 10. Jumbled words | 0.70 [0.55–0.84] | 0.75 [0.61–0.89] |
| 11. Book | 0.81 [0.71–0.91] | 0.76 [0.63–0.90] |
| 12. Familiar tasks | 0.86 [0.78–0.94] | 0.72 [0.57–0.87] |
| 13. Staying focused | 0.74 [0.61–0.87] | 0.85 [0.76–0.94] |
| 14. Learning new things | 0.77 [0.65–0.89] | 0.59 [0.39–0.78] |
| 15. Speaking fast | 0.80 [0.70–0.91] | 0.83 [0.74–0.93] |
| 16. Doing things quickly | 0.72 [0.59–0.86] | 0.78 [0.66–0.91] |
| 17. Changes in routine | 0.85 [0.77–0.93] | 0.84 [0.75–0.94] |
| 18. What people mean | 0.85 [0.76–0.93] | 0.73 [0.58–0.87] |
| 19. How other people feel | 0.81 [0.71–0.91] | 0.86 [0.78–0.95] |
| 20. Group conversation | 0.86 [0.78–0.94] | 0.87 [0.79–0.95] |
| **SCoRS Total Score** | **0.87 [0.80–0.94]** | **0.91 [0.86–0.97]** |
| SCoRS Global Rating | 0.63 [0.46–0.80] | 0.61 [0.42–0.80] |

CI, confidence interval; ICC, interclass correlation; SCoRS, the Schizophrenia Cognition Rating Scale.

#### Supplementary Table 3. Distribution of the SCoRS Total Scores and Global Rating Scores.

| **Mode** | **n** | **Mean** | **SD** | **Median** | **IQR** | | **Range** | |
| --- | --- | --- | --- | --- | --- | --- | --- | --- |
|  |  |  |  |  | **25** | **75** | **Min** | **Max** |
| **SCoRS Total Score (20–80)** | | | | | | | | |
| Live | 44 | 42.0 | 10.54 | 42.0 | 35.0 | 49.0 | 23 | 68 |
| VR1 | 44 | 41.0 | 10.19 | 41.0 | 33.5 | 48.5 | 22 | 63 |
| VR2 | 44 | 41.2 | 10.19 | 42.0 | 33.5 | 48.0 | 22 | 66 |
| All | 132 | 41.4 | 10.24 | 42.0 | 34.0 | 48.5 | 22 | 68 |
| **SCoRS Global Rating Score (1–10)** | | | | | | | | |
| Live | 44 | 4.7 | 1.55 | 5.0 | 3.0 | 6.0 | 2 | 8 |
| VR1 | 44 | 4.3 | 1.53 | 4.0 | 3.0 | 5.0 | 1 | 8 |
| VR2 | 44 | 4.5 | 1.76 | 4.0 | 3.0 | 6.0 | 2 | 8 |
| All | 132 | 4.5 | 1.61 | 4.0 | 3.0 | 6.0 | 1 | 8 |
| Note: Higher values denote greater difficulties. | | | | | | | | |

IQR, interquartile range; SCoRS, the Schizophrenia Cognition Rating Scale; SD, standard deviation; VR1/2, video recording 1 or 2.

#### Supplementary Table 4. Patient, informant, and interviewer eligibility criteria.

| ***Patient and informant inclusion criteria*** |
| --- |
| - Patients must be capable of providing signed and dated written informed consent in accordance with ICH Harmonized Tripartite Guideline for Good Clinical Practice (ICH-GCP) and local legislation. - Male or female patients who are 18–50 years (inclusive) of age at time of consent. - Diagnosis of schizophrenia utilizing DSM-V™ with the following clinical features: Outpatient, clinically stable and in the residual (non-acute) phase of their illness. - No hospitalization or increase in level of psychiatric care due to worsening of schizophrenia within 12 weeks prior to enrollment. - PANSS score at screening: items P1, P3-P6 ≤5 and item P2 and P7 ≤4 at Visit 1. - Patients should have functional impairment in day-to-day activities such as difficulties following conversation or expressing themselves, difficulties to stay focused, difficulties to remember instructions, what to say, or how to get to places, per investigator judgement. - Patients must have been maintained on their current antipsychotic treatment (minimum one and maximum two antipsychotics; no clozapine) for at least 12 weeks and on their current dose for at least 35 days prior to enrollment. - Patients must have a study partner (informant), defined as any person either private or professional (e.g., study nurse, social worker) who knew the patient well, and interacted with the patient on regular basis. - The informant must interact with the subject for a minimum of 1 hour per week and, preferably, at least two times a week. At least one interaction per week should be in person. - The informant must have achieved an educational level of at least 8th grade. - Professional informants (e.g., study nurse, social worker) were permitted even if not involved in the administration of any of the study activities. - Patients must, in the investigator’s opinion, exhibit reliability and physiologic capabilities (e.g., sufficient hearing, vision) to take part in the SCoRS interview and have attained an educational level of at least 8th grade. - Patients and their informant must be fluent in English. - Patients and their informant must not be currently participating in a clinical trial. - Patients must not have taken part in a SCoRS interview within the past 3 months. |
| ***Patient and informant exclusion criteria*** |
| - Participants with a current DSM-V diagnosis other than schizophrenia, including, but not limited to, bipolar disorder, schizoaffective disorder, and major depressive disorder. - Cognitive impairment due to developmental, neurological (e.g., epilepsy, stroke), or other disorders, including head trauma or patients with dementia. |
| ***Interviewer (rater) inclusion criteria*** |
| - Fully qualified and trained to conduct the SCoRS interviews and provide interviewer ratings, as confirmed by WCG. - Willing and able to score 2–4 patients via live interviews (i.e., speak with 2–4 patients and their unique informants) and score 4–8 patients via recorded interviews (i.e., watch interviews with 4–8 patients and their unique informants), resulting in each interviewer providing the SCoRS data for 6–12 patients overall. - Be able to conduct patient and informant interviews within 7 days of each other. In addition, the SCoRS ratings had to be completed and submitted within 48 hours of completing the second interview (e.g., once interviews are complete for each dyad). |

DSM-V, Diagnostic and Statistical Manual of Mental Disorders, 5^th^ edition; ICH, International Council for Harmonization of technical requirements for pharmaceuticals for human use; PANSS, Positive and Negative Syndrome Scale; SCoRS, the Schizophrenia Cognition Rating Scale; WCG, Western Institutional Review Board-Copernicus Group.

#### Supplementary Table 5. SCoRS rater requirements and training prior to the first interview with patients or informants, and video-recording standards

| **SCoRS rater requirements and training** |
| --- |
| Interviewers (raters) were trained to maintain high quality standards for interviewing in accordance with real-world practices for SCoRS administration. Interviewers (raters) were required to be qualified clinicians; the rater credentials and experience were monitored by WCG, the SCoRS instrument license holder. Interviewers (raters) were required to be medical experts (e.g., psychiatrist, psychologist, advanced degree in psychology or related psychological services) with a minimum educational requirement of Bachelor’s degree (or equivalent) and a minimum of one year of experience working with the schizophrenia population.  Prior to the first interview with patients or informants, the following applied:   1. Qualified raters were asked to complete the following: an assessment overview, review a set of pre-recorded interviews in the WCG training portal and submit ratings for the interviews to WCG, a certification session with a WCG certification specialist. 2. WCG reviewed ratings submitted for the pre-recorded interviews and compared them with the WCG gold standard scores. 3. Raters were required to participate in a one-to-one/group certification session and demonstrate their ability to administer and score the assessment according to the administration guidelines. If the rater was unable to demonstrate sufficient understanding of the administration and scoring of the assessment, then a follow-up call may have been required depending on the rater’s performance. 4. If the rater completed all the steps above, they were considered certified. 5. A rater could be considered exempt from training for the SCoRS if the rater had been certified by WCG on the assessment and administered the assessment within the last year and there were no concerns about the quality of the assessments that the rater has administered. In these cases, the rater had to submit a practice upload using WCG Pathway. Following this, the rater would be considered certified. |
| **Video-recording standards and training** |
| To ensure consistency and stability of the video recordings, sites were supplied with an adjustable, foldable smartphone holder, an extended-length smartphone power cable, and a USB-to-ethernet cable for ensuring good internet bandwidth for the CIRP VS tablet. For the smartphone, connecting to Wi-Fi was required to enable the app-to-app video stream and recording – a Wi-Fi hotspot with a data plan to ensure good bandwidth was provisioned per site. Devices were pre-installed with CIRP VS, having undergone installation qualification testing and QC, and access to non-CIRP tablet functionality will be restricted as far as possible.  Training on the set-up and use of the device was provided. Device functionality, including the quality of recording, was confirmed once at site so any troubleshooting (such as firewall access) could be resolved before the site was able to actively conduct subject interviews. Technical support was provided to sites to troubleshoot any issues which may arise. |

SCoRS, the Schizophrenia Cognition Rating Scale; WCG, WIRB Copernicus Group.
